# Supplementary material for: Acoustic ejection mass spectrometry empowers ultra-fast protein biomarker quantification
Source: Nat Commun. 2024 Jun 15;15:5114. doi: 10.1038/s41467-024-48563-z (PMC11180209; doi:10.1038/s41467-024-48563-z)
Supplement: Supplementary file 1 — Supplementary Information [file 41467_2024_48563_MOESM1_ESM.pdf]

Supplementary Information

Acoustic Ejection Mass Spectrometry Empowers Ultra-Fast Protein Biomarker Quantification

Bart Van Puyvelde [1,2]*, Christie Hunter [3]*, Maxim Zhgamadze [2], Sudha Savant [4], Y. Oliver Wang [2], Esthelle Hoedt [2], Koen Raedschelders [2], Matt Pope [5], Carissa A. Huynh [6] V. Krishnan Ramanujan [6], Warren Tourtellotte [6], Morteza Razavi [5], N. Leigh Anderson [5], Geert Martens [7], Dieter Deforce [1], Qin Fu [2], Maarten Dhaenens [1°], Jennifer E. Van Eyk [2°]

[1] ProGenTomics, Laboratory of Pharmaceutical Biotechnology, Ghent University, 9000 Ghent, Belgiumi7

[2] Advanced Clinical Biosystems Research Institute, Smidt Heart Institute, Cedars-Sinai Medical Center, Los Angeles, CA, 90048, USA

[3] SCIEX, Redwood City, CA, 94065, USA

[4] Beckman Coulter Life Sciences

[5] SISCAPA Assay Technologies, Inc., Box 53309, Washington, DC 20009, United States of America and Victoria, BC Canada

[6] Cedars Sinai Biobank & Research Pathology Resource, Cedars-Sinai Medical Center, Los Angeles, CA, 90048, USA

[7] AZ Delta Medical Laboratories, AZ Delta General Hospital, 8800 Roeselare, Belgium

* These authors contributed equally

° These authors jointly supervised the work

Corresponding authors:

Maarten Dhaenens – Maarten.Dhaenens@ugent.be

Jennifer Van Eyk – Jennifer.VanEyk@cshs.org

Short title: AEMS for peptide detection

Keywords: Mass Spectrometry, SARS-CoV-2, Biomarkers, Acoustic Ejection Mass Spectrometry, Inflammation

Supplementary Figures

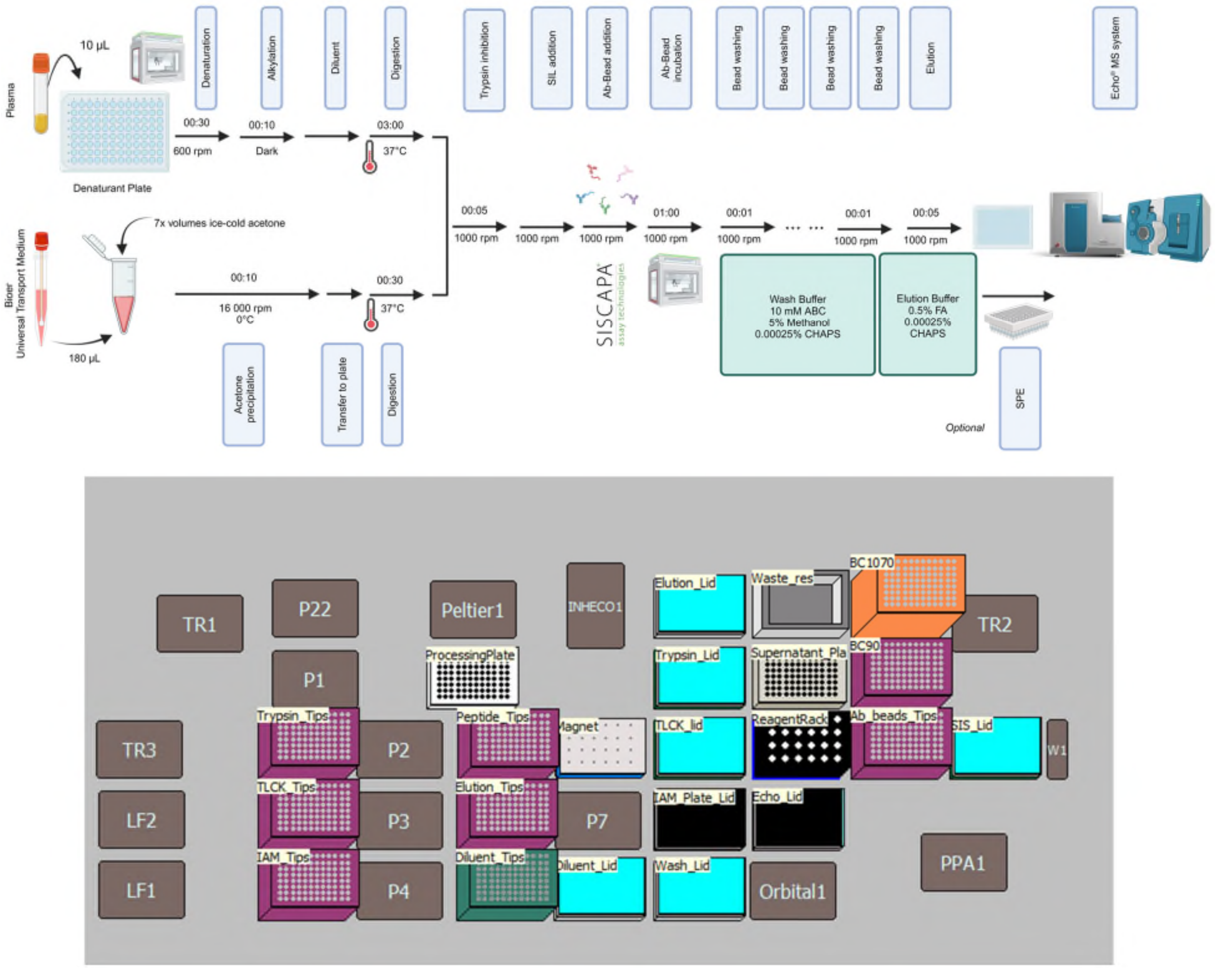

Supplementary Figure 1. Representation of automated SISCAPA workflow using the Biomek i7 workstation. (Top) Schematic representation of the automated SISCAPA workflow for the 10-plex APR (Top – Plasma) and SARS-CoV-2 Nucleocapsid (NCAP) (Lower – Nasopharyngeal swab) detection. The Biomek i7 workstation was only used for NCAP peptide detection once antibody-beads were added to the samples. Figure created with BioRender.com. (Bottom) In the overview, the Inheco1 incubator was used for trypsin digestion, while the Peltier1 cooler was used to maintain trypsin at 4°C prior to digestion initiation and the Orbital1 orbital shaker was used for magnetic bead resuspension. The positive pressure apparatus (PPA) was not utilized in the SISCAPA protocol. Additionally, TR represents the three trash bins and LF represents labware feeders. Supplementary Figure 1, created with BioRender.com, released under a Creative Commons Attribution-NonCommercial-NoDerivs 4.0 International license.

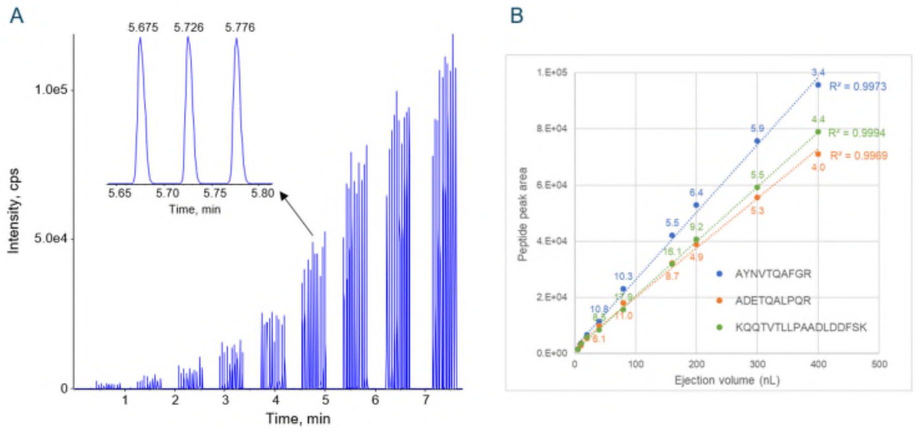

Supplementary Figure 2. Linearity of acoustic ejection with increasing droplet number. Each sample ejection consists of a stream of rapidly ejected 2.5 nL droplets which creates a single peak. Increasing the number of droplets ejected per sample and hence the total ejection volume increases the peptide peak area and improves the sensitivity of the assay. A) 10 replicate ejections of SARS-CoV-2 AYNVTQAFGR peptide (5 fmol/µL) were measured using increasing numbers of droplets per ejection. Inset shows the peak shape of a typical ejection peak. B) Linearity between increasing total ejection volume and peak area for the three SARS-CoV-2 peptides is very good from 5 to 400 nL of peptide ejected for the NCAP peptides AYNVTQAFGR (blue), ADETQALPQR (orange) and KQQTVTLLPAADLDDFSK (green). In addition, reproducibility of peak area calculated as coefficient of variation (%CV) is annotated above each datapoint.

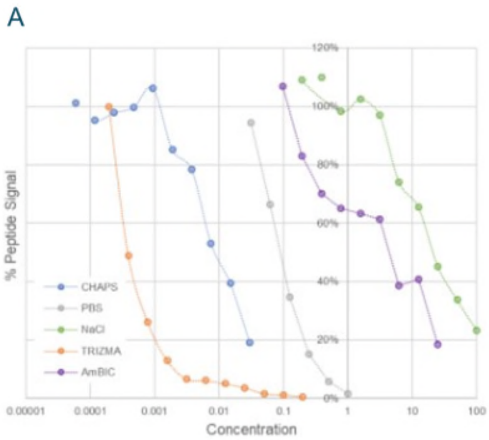

B

Additive	Starting [conc]	Max [conc] tolerated
CHAPS	0.03%	0.01%
PBS	1x	0.06250x dilution
NaCl	100 mM	12.5 mM
TRIZMA	0.2 M	0.391 mM
AmBic	100 mM	6.25 mM

Supplementary Figure 3. Assessing the impact of additives in the sample well on peptide ejection signal. A) Increasing amounts of various additives were mixed with a constant amount of ADETQALPQR peptide, ejected in a series of sample wells. The impact of peptide peak area and ejection peak shape were evaluated. B) The table summarizes the starting concentration of the 1:2 dilution series and the maximum tolerated concentration for each buffer component (CHAPS – blue, PBS – grey, NaCl – green, TRIZMA – orange, Ammonium bicarbonate (AmBic) – purple) tested, which was set at ~50% of original peptide signal. Concentrations above these values should be avoided.

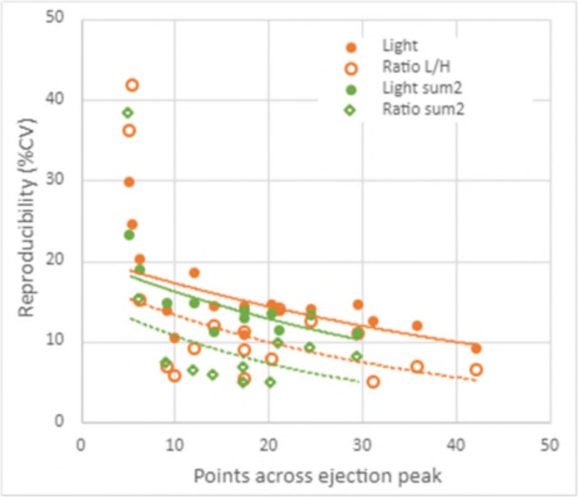

Supplementary Figure 4. Optimization of MRM method for AEMS peptide analysis. As with LC-MS analysis using SRM/MRM, the used dwell time should be maximized where possible and the points across the peak should be optimized to obtain the best measurement reproducibility. A matrix of methods using 2, 4 or 6 MRMs with dwell times ranging from 3 – 100 ms were made for the LAEGFPLPLLK peptide from LBP, then 18 replicate ejections were measured using 160 nL ejection volumes for each. Peak area reproducibility (single best MRM (solid orange) or sum of MRMs (solid green)) was plotted vs. the observed points across the ejection peak which highlights that peak sampling of 10 points across the peak or more are needed for maintaining high reproducibility. Comparing the L/H peak area ratios vs points across the peak showed a similar trend, with slightly better reproducibility than peak area (single best MRM L/H peak area ratio (open orange) or sum of MRMs L/H peak area ratio (open green). The final optimized method consisted of 4 MRMs per method with a 10 ms dwell time each, plus the additional CHAPS MRM with a 3 ms dwell time, and a pause time of 3 ms between each MRM for a total cycle time 58 ms.

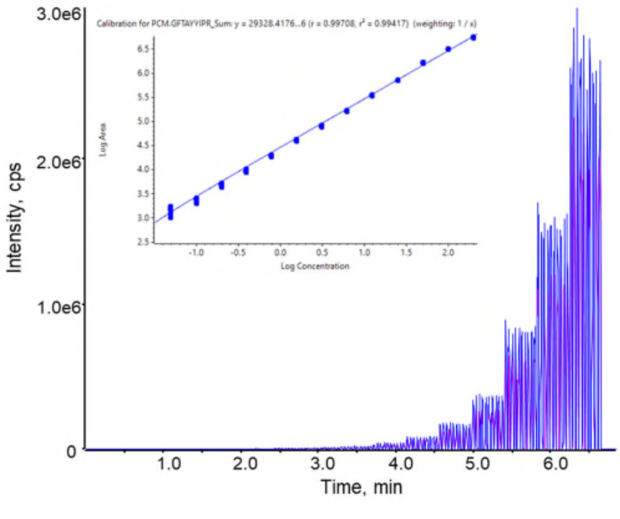

Supplementary Figure 5. Determining lower limits of quantification (LLOQs) for peptides using AEMS. Calibration curves were generated using 20 standard peptides in a simple matrix (0.5% formic acid, 0.00025% CHAPS). Lower limits of quantification were determined as the lowest concentration where the %CV across 10 replicates was < 20% and the accuracy was between 80-120%. Data shown is for the peptide GFTAYYIPR from PepCalMix.

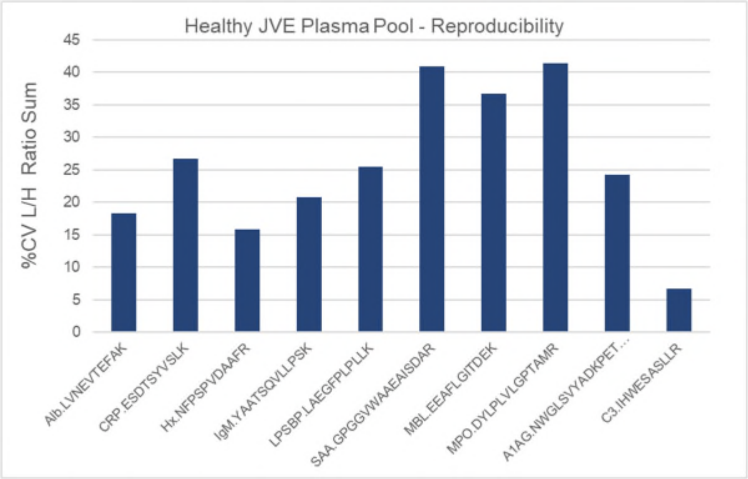

Supplementary Figure 6. Reproducibility of replicate sample processing of a plasma pool. Variability of L/H ratios across 19 replicate wells of pooled healthy plasma for all ten APR peptides across the three sample plates.

Protein	Light peptide peak area minimum threshold	Heavy peptide peak area minimum threshold
Alb.LVNEVTEFAK	2000	2000
CRP.ESDTSYVSLK	200	200
Hx.NFPSPVDAAFR	2000	2000
IgM.YAATSQVLLPSK	1000	1000
LBP.LAEGFPLPLLK	1000	1000
SAA.GPGGVWAAEAISDAR	200	200
MBL.EEAFLGITDEK	2000	2000
MPO.DYLPLVLGPTAMR	1500	1500
A1AG.NWGLSVYADKPETTK	1000	1000
C3.IHWESASLLR	1000	1000

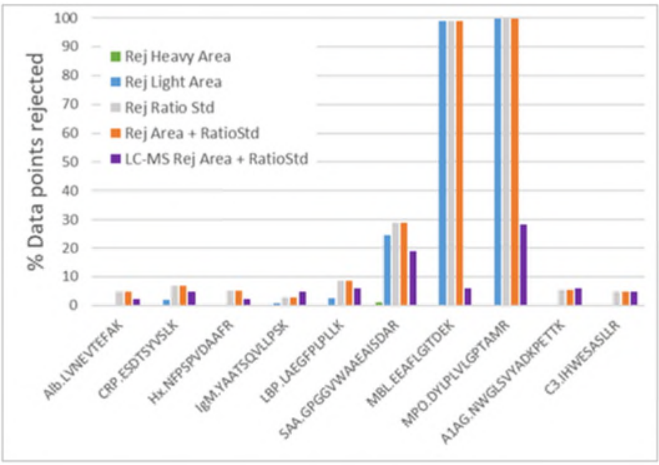

Supplementary Figure 7. Impact of outlier rejection on APR dataset. Lower limits of quantification for the light peptides were determined in simple matrix and the peptide areas (sum of the peak areas for both MRM transitions) were determined. A) Peak areas minimums were set for each peptide and data points were removed from the dataset if they did not meet this criterion. Note for CRP and SAA where the endogenous levels of light peptide in healthy individuals are known to be very low, we set the minimum peak area threshold lower in order to not reject all healthy data points. B) Outlier rejection was performed by setting the maximum allowed L/H peak area ratio difference between the two peptide fragments monitored to be the average fragment ratio difference plus 2-sigma and by setting a minimum summed peak area filter for the light and heavy peptide. Outlier rejection had low impact on proteins with substantial light (Blue) peptide signal with only 2-10% of datapoints being removed, which contrasts with the very low abundant proteins e.g., MBL and MPO with almost 100% data point removal. The same rejection strategy was applied to the LC-MS data (Purple), note the improved detection of the lower abundant proteins, SAA, MBL and MPO due to the lower flow chromatography and higher injection amount. Samples were excluded based on specific criteria: those with too low light (light blue)/heavy (light green) peptide signal, those exhibiting a high difference in L/H-ratio (depicted in light grey), or samples not meeting both criteria (highlighted in orange).

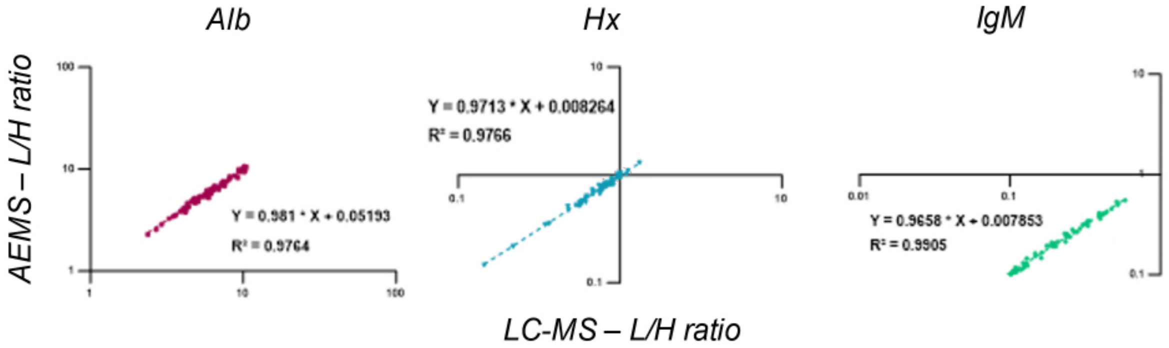

Supplementary Figure 8. Correlation of measured AEMS L/H peptide ratios with LC-MS data. The ratios measured for Albumin (Alb), Hemopexin (Hx) and Immunoglobulin M (IgM) by LC-MS were very similar to the ratios determined using the Echo MS system (n = 69).

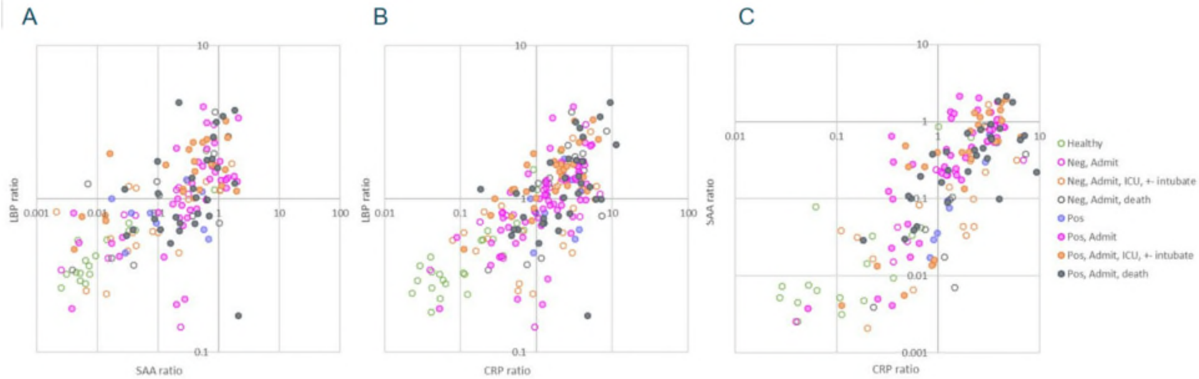

Supplementary Figure 9. Expression correlations between proteins during acute phase response. In samples from individuals presenting at the hospital with COVID like symptoms, protein levels for CRP, SAA and LBP increase. Here we see a correlation in expression between A) SAA vs LBP levels, B) CRP vs LBP levels and between C) CRP and SAA levels.

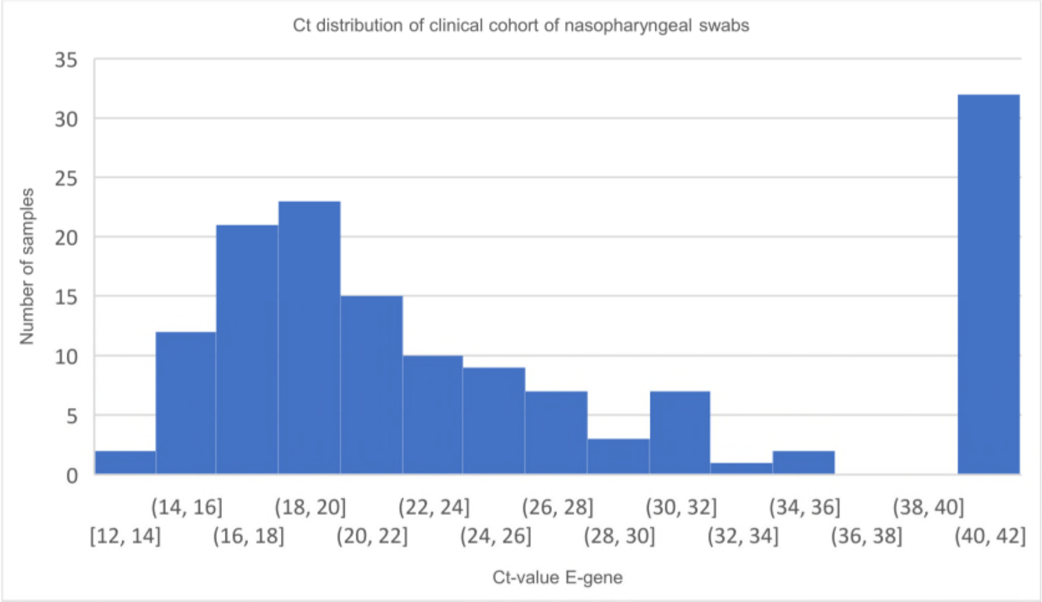

Supplementary Figure 10. Ct distribution of nasopharyngeal swab samples. Bar graph illustrating the distribution of samples per Ct-value, showing the frequency of samples across different Ct ranges

A

Protein	Light peptide peak area minimum threshold	Heavy peptide peak area minimum threshold
SARSCoV2.AYNVTQAFGR	1000	1000
SARSCoV2.ADETQALPQR	1500	1500
SARSCoV2.KQQTVTLLPAADLDDFSK	1500	1500

B

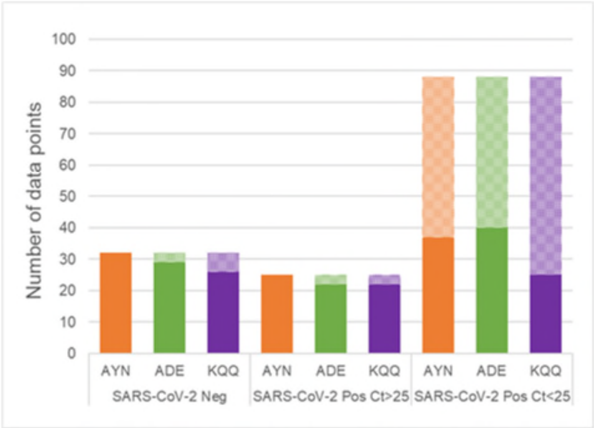

Supplementary Figure 11. Impact of outlier rejection strategy for SARS-CoV-2 NCAP peptides. Similarly to the outlier rejection strategy used for the APR assay, we used calibration curves in Universal Transport Medium (UTM) to determine the peak areas for each peptide (AYNVTQAFGR – orange, ADETQALPQR – light green, KQQTVTLLPAADLDDFSK – purple) at their LLOQ. A) Peak area minimums were defined for each peptide and data points were removed from the dataset if they did not meet this criterion. B) Outlier rejection was performed by a 2sigma average difference maximum in fragment area ratios observed across the dataset and by setting a minimum summed peak area filter for the light and heavy peptide. The samples were divided into three groups based on COVID status (Negative, Positive E-gene Ct>25 and E-gene positive Ct<25, (Figure 5)) and the number of rejected samples out of the total samples within each group was determined (solid fill). For these three peptides, the majority of rejected data points were from the SARS-CoV-2 negative samples and the samples where the E-gene Ct >25.

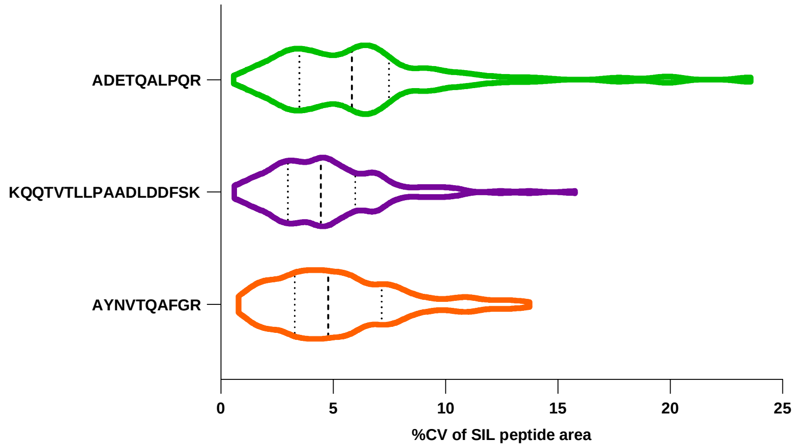

Supplementary Figure 12. Reproducibility of SARS-CoV-2 SIL peptide areas. The reproducibility of the SIL peptide measured in triplicate for each enriched sample (n = 145) was found to be very good, with average %CV values across the sample set of 5.4, 6.4 and 4.8% for the peptides AYNVTQAFGR (orange), ADETQALPQR (light green) and KQQTVTLLPAADLDDFSK (purple), respectively. Data was subjected to the outlier rejection strategy and rejected data points were not plotted.

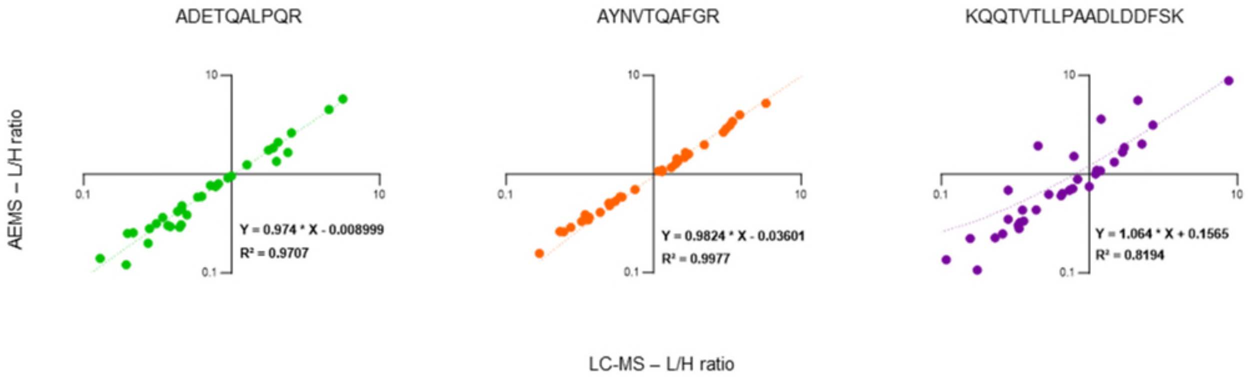

Supplementary Figure 13. Correlation of measured AEMS peptide ratios with LC-MS data for SARS-CoV-2 NCAP peptides. The ratios measured by LC-MS were very similar to the ratios determined using the Echo MS system (n = 63). Scatter for the KQQTVTLLPAADLDDFSK (purple) peptide was higher because the chromatographic behavior of this peptide was poorer than the other two peptides (AYNVTQAFGR – orange, ADETQALPQR – light green). Points in the bottom left quadrant had very low endogenous peptide levels, below the detection level of the method and were typically from the COVID negative individuals. Data was subjected to the outlier rejection strategy and rejected data points were not plotted.

Supplementary Tables

Supplementary Table 1. Composition of the 10-plex SIL mixture. Proteotypic peptides comprised of an amino acid sequence unique to each target protein were mixed at various concentrations to reflect the abundance of the proteins expected in plasma and also to ensure adequate SIL peptide areas were obtained post-capture. The known roles of each protein are included including the protein's role as either a positive acute phase reactants (APR+) and negative acute phase reactants (APR-).

Peptide sequence	Protein (abbreviation)	SIL concentration (fmol/µL)	Biological role*
LVNEVTEFAK	Albumin (Alb)	5000	APR-
NWGLSVYADKPETTK	Alpa-1 Acid Glycoprotein (A1AG)	200	APR+
IHWESASLLR	Complement component C3 (C3)	250	Complement, APR+
ESDTSYVSLK	C-reactive protein (CRP)	1010	Innate immunity, APR+
NFPSPVDAAFR	Hemopexin (Hx)	1000	Fe metabolism, APR+
YAATSQVLLPSK	Immunoglobulin M (IgM)	1000	Immune response, APR±
LAEGFPLPLLK	Lipopolysaccharide Binding Protein (LBP)	10	Innate immunity, APR+
EEAFLGITDEK	Mannose-binding lectin (MBL)	10	Innate immunity, APR+
DYLPLVLGPTAMR	Myeloperoxidase (MPO)	10	Neutrophil count
GPGGVWAAEAISDAR	Serum Amyloid A (SAA)	510	Innate immunity, APR+

*Role of protein in acute phase response adapted from Anderson et al. (2020) (18)

Supplementary table 2. MRM transitions used for analysis of the 10 acute phase response proteins. Quadrupole resolution for both Q1 and Q3 was set to unit.

Peptide	Q1	Q3	DP	CE	CXP
A1AG.NWGLSVYADKPETTK.+3y13+2.light	570.29	704.87	80	25.4	15
A1AG.NWGLSVYADKPETTK.+3y13+2.heavy	572.96	708.88	80	25.4	15
A1AG.NWGLSVYADKPETTK.+3y11+2.light	570.29	619.81	90	28.4	15
A1AG.NWGLSVYADKPETTK.+3y11+2.heavy	572.96	623.82	90	28.4	15
Alb.LVNEVTEFAK.+2y8.light	575.31	937.46	70	24.2	15
Alb.LVNEVTEFAK.+2y8.heavy	579.32	945.48	70	24.2	15
Alb.LVNEVTEFAK.+2y6.light	575.31	694.38	80	27.2	15
Alb.LVNEVTEFAK.+2y6.heavy	579.32	702.39	80	27.2	15
C3.IHWESASLLR.+2y7.light	606.33	775.43	90	34.7	15
C3.IHWESASLLR.+2y7.heavy	611.33	785.44	90	34.7	15
C3.IHWESASLLR.+3y6.light	606.33	646.39	90	34.7	15
C3.IHWESASLLR.+3y6.heavy	611.33	656.40	90	34.7	15
CRP.ESDTSYVSLK.+2y6.light	564.78	696.39	60	26.7	15
CRP.ESDTSYVSLK.+2y6.heavy	568.78	704.41	60	26.7	15
CRP.ESDTSYVSLK.+2y5.light	564.7	609.36	80	23.7	15
CRP.ESDTSYVSLK.+2y5.heavy	568.78	617.38	80	23.7	15
Hx.NFPSPVDAAFR.+2y9.light	610.81	959.50	70	25.9	15
Hx.NFPSPVDAAFR.+2y9.heavy	615.81	969.50	70	25.9	15
Hx.NFPSPVDAAFR.+2y9+2.light	610.81	480.25	60	25.9	15
Hx.NFPSPVDAAFR.+2y9+2.heavy	615.81	485.26	60	25.9	15
IgM.YAATSQVLLPSK.+2y10.light	639.36	1043.61	70	30.3	15
IgM.YAATSQVLLPSK.+2y10.heavy	643.37	1051.62	70	30.3	15
IgM.YAATSQVLLPSK.+2y9.light	639.36	972.57	90	30.3	15
IgM.YAATSQVLLPSK.+2y9.heavy	643.37	980.59	90	30.3	15
LBP.LAEGFPLPLLK.+2y9.light	599.37	1013.60	70	25.4	15
LBP.LAEGFPLPLLK.+2y9.heavy	603.37	1021.62	70	25.4	15
LBP.LAEGFPLPLLK.+2y6.light	599.37	680.47	70	25.4	15
LBP.LAEGFPLPLLK.+2y6.heavy	603.37	688.49	70	25.4	15
MBL.EEAFLGITDEK.+2y6.light	626.31	662.34	70	30.3	15
MBL.EEAFLGITDEK.+2y6.heavy	630.32	670.35	70	30.3	15
MBL.EEAFLGITDEK.+2y7.light	626.31	775.42	90	30.3	15
MBL.EEAFLGITDEK.+2y7.heavy	630.32	783.43	90	30.3	15
MPO.DYLPLVLGPTAMR.+2y10.light	723.40	1054.61	70	37.4	15
MPO.DYLPLVLGPTAMR.+2y10.heavy	728.40	1064.62	70	37.4	15
MPO.DYLPLVLGPTAMR.+2y10+2.light	723.40	527.81	60	28.4	15
MPO.DYLPLVLGPTAMR.+2y10+2.heavy	728.40	532.81	60	28.4	15
SAA.GPGGVWAAEAISDAR.+2y10.light	728.86	1089.53	100	40.7	15
SAA.GPGGVWAAEAISDAR.+2y10.heavy	733.87	1099.54	100	40.7	15
SAA.GPGGVWAAEAISDAR.+2y9.light	728.86	903.45	110	40.7	15
SAA.GPGGVWAAEAISDAR.+2y9.heavy	733.87	913.46	110	40.7	15

CHAPS	615.40	448.40	100	40.0	15

Supplementary table 3: MRM transitions used for analysis of the 3 peptides from NCAP_SARS2. Quadrupole resolution for both Q1 and Q3 was set to unit.

Peptide	Q1	Q3	DP	CE	CXP
CovID19NCAP.ADETQALPQR.+2y3.light	564.79	400.23	95	**26.2**	**20**
CovID19NCAP.ADETQALPQR.+2y3.heavy	569.79	410.24	95	26.2	20
CovID19NCAP.ADETQALPQR.+2y5.light	564.79	584.35	95	30.2	20
CovID19NCAP.ADETQALPQR.+2y5.heavy	569.79	594.36	95	30.2	20
CovID19NCAP.AYNVTQAFGR.+2y6.light	563.79	679.35	65	26.2	20
CovID19NCAP.AYNVTQAFGR.+2y6.heavy	568.79	689.36	65	26.2	20
CovID19NCAP.AYNVTQAFGR.+2y7.light	563.79	778.42	65	30.2	20
CovID19NCAP.AYNVTQAFGR.+2y7.heavy	568.79	788.43	65	30.2	20
CovID19NCAP.KQQTVTLLPAADLDDFSK.+3y10++.light	664.02	539.76	80	28.2	20
CovID19NCAP.KQQTVTLLPAADLDDFSK.+3y10++.heavy	666.69	543.76	80	28.2	20
CovID19NCAP.KQQTVTLLPAADLDDFSK.+3y10.light	664.02	1078.51	80	28.2	20
CovID19NCAP.KQQTVTLLPAADLDDFSK.+3y10.heavy	666.69	1086.52	80	28.2	20
CHAPS	615.40	448.40	100	40.0	15

Supplementary Table 4. Gradient used for LC-MS analysis of APR and NCAP_SARS2 samples. A 2.5 min gradient from 5-27% mobile phase B was used for elution of peptides, then two rapid washes were performed for a total LC run time of 6.5 mins (see Supplementary Table 4). Sample injection protocol was 2 mins for a total sample analysis time of 8.5 mins.

Time (min)	Mobile phase A (%)	Mobile phase B (%)	Flow rate (µL/min)
0	95	5	10
2.5	73	27	10
3	30	70	10
3.5	95	5	10
4	60	40	10
4.5	95	5	10
6.5	95	5	10

Supplementary Table 5. Percent coefficient of variation (%CV) for the automated sample preparation, the AEMS analysis, and the total workflow. A pool and split strategy using 16 processed wells was used to evaluate reproducibility.

	**Alb**	**A1AG**	**C3**	**CRP***	**Hx**	**IgM**	**LBP***	**MBL***	**MPO***	**SAA***
**MS CV (%)**	3.76	7.16	5.66	9.99	3.79	4.41	5.38	6.73	9.43	6.18
**Biomek CV (%)**	4.82	5.46	7.16	2.49	5.52	2.03	3.28	5.49	7.24	2.97
**Total CV (%)**	6.11	9.01	9.13	10.3	6.69	4.86	6.30	8.68	11.89	6.86

Supplementary Table 6. Lower limits of quantification (LLOQs) for a range of peptides using AEMS. The lower limits of quantification were determined for the 20 standard peptides and summarized here, including the %CV, %accuracy at the LLOQ and the R^2 value for the curve. Experiment was repeated on two Echo MS systems to ensure repeatable results. One peptide (SGGLLWQLVR) did not behave well in this assay, this peptide is very hydrophobic and also not well behaved in LC-MS.

	Instrument 1				Instrument 2			
**Peptide**	**LLOQ (fmol/µL)**	**% CV**	**% Accuracy**	**R2**	**LLOQ (fmol/µL)**	**% CV**	**% Accuracy**	**R2**
PCM.AETSELHTSLK	0.390	19.6	88.8	0.997	0.390	19.1	82.2	0.994
PCM.GAYVEVTAK	0.390	8.8	101.5	0.998	0.100	8.4	97.8	0.999
PCM.IGNEQGVSR	0.781	13.4	86.7	0.997	0.200	10.6	105.7	0.995
PCM.LVGTPAEER	0.195	14.3	97.6	0.999	0.100	7.3	93.3	0.999
PCM.LDSTSIPVAK	0.098	18.7	114.5	0.999	0.100	14.9	99.1	0.996
PCM.AGLIVAEGVTK	0.049	17.0	105.4	0.999	0.050	11.2	114.7	0.997
PCM.LGLDFDSFR	0.781	5.2	118.6	0.994	0.390	3.6	112.6	0.982
PCM.GFTAYYIPR	0.098	5.8	117.1	0.999	0.200	5.1	116.1	0.996
PCM.SGGLLWQLVR	- -	- -	- -	- -	- -	- -	- -	- -
PCM.AVGANPEQLTR	0.098	17.6	94.7	0.997	0.050	13.2	85.3	0.989
PCM.SAEGLDASASLR	0.195	12.3	81.2	0.998	0.050	13.8	103.8	0.997
PCM.VFTPLEVDVAK	0.195	18.7	93.6	0.999	0.200	7.6	113.1	0.991
PCM.VGNEIQYVALR	0.195	14.9	104.8	0.999	0.200	4.3	111.1	0.995
PCM.YIELAPGVDNSK	0.977	12.9	81.0	0.997	0.100	18.6	104.6	0.996
PCM.DGTFAVDGPGVIAK	0.097	7.5	105.4	0.998	0.200	9.2	116.4	0.996
PCM.YDSINNTEVSGIR	0.390	7.1	96.9	0.998	0.390	17.1	80.2	0.990
PCM.SPYVITGPGVVEYK	0.195	18.6	102.1	0.996	0.390	10.2	109.3	0.982
PCM.ALENDIGVPSDATVK	0.098	18.5	80.0	0.998	0.200	4.4	119.3	0.985
PCM.AVYFYAPQIPLYANK	1.562	19.6	108.8	0.987	0.780	5.9	117.9	0.902
PCM.TVESLFPEEAETPGSAVR	3.125	3.8	115.3	0.991	0.780	7.4	102.7	0.985
**Average**	**0.52**	**13.37**	**99.69**	**0.997**	**0.26**	**10.1**	**104.47**	**0.988**

Supplementary Table 7. Lower limits of quantification (LLOQs) for 10 APR peptides using AEMS. Lower limits of quantification were determined as the lowest concentration where the %CV across 5 replicates was < 20% and the accuracy was between 80-120%.

	Instrument 1			
**Peptide**	**LLOQ (fmol/uL)**	**%CV**	**%Accuracy**	**R2**
A1AG.NWGLSVYADKPETTK	0.610	10.3	118.3	0.991
Alb.LVNEVTEFAK	0.310	17.5	89.4	0.993
C3.IHWESASLLR	1.220	18.1	118.8	0.997
CRP.ESDTSYVSLK	0.610	9.2	87.2	0.998
Hx.NFPSPVDAAFR	0.150	14.3	96.1	0.996

IgM.YAATSQVLLPSK	0.610	21.8	81.5	0.998
LBP.LAEGFPLPLLK	0.610	9.7	106.9	0.989
MBL.EEAFLGITDEK	0.310	14.8	84.7	0.995
MPO.DYLPLVLGPTAMR	0.610	8.4	107.3	0.992
SAA.GPGGVWAAEAISDAR	0.610	4.5	129.9	0.994
**Average**	**0.57**	**12.85**	**102.00**	**0.994**

Supplementary Table 8. Classification of cohort samples based on COVID status and disease severity. Subjects included in the Covid-19 cohort were 51% male and 49% female, with a median age of 70 (range 25-102 years old). The median age of the Healthy individuals was 43, with a male/female distribution of 17%/83%. For the healthy pool workflow replicates, gender pooled plasma from BioIVT was used.

**Disease Status**	**# of samples in cohort**
Healthy pool (workflow replicates)	19
Healthy individuals	27
COVID Negative, Admitted	13
COVID Negative, Admitted, ICU, ±intubate	26
COVID Negative, Admitted, death	17
COVID Positive	12
COVID Positive, Admitted	70
COVID Positive, Admitted, ICU, ±intubate	36
COVID Positive, Admitted, death	47
**Total**	**267**

Supplementary Methods

Automated sample preparation protocol

The automated sample preparation was performed on the Biomek i7 dual hybrid workstation from Beckman Coulter Life Sciences. Briefly, 17 µL of a 'denaturation mix' (9 M urea, 0.05 M TCEP and 0.2 M Trizma) was aliquoted into each V-shaped well of a 96 well plate (Prod. No P-96-450V-C-S; Axygen). The mixture was then incubated overnight in a dry incubator at 37°C, allowing the mixture to dry in the wells. The next morning, 10 µL of human plasma (BiolVT, Westbury NY, USA or from the CSMS cohort) was added to each well followed by a 30-min incubation on a ThermoMixer F (Eppendorf, Hamburg, Germany) at room temperature with vigorous mixing at 600 rpm. Although sample addition is automatable, here we manually transferred the plasma to prevent sample loss from aliquoting. All subsequent steps were performed with a Biomek i7. Cysteine alkylation was performed by adding 10 µL of a 0.05M IAA solution, followed by an incubation of 10 min at room temperature in the dark (dark lid covering the plate). Following dilution with 115 µL of 0.2 M Trizma buffer to reach a final urea concentration of 1 M, 10 µL of 7.3 mg/mL trypsin (Worthington) in 10 mM HCl was added. The 96-well plate was then incubated for 3 hours at 37°C on a shaking incubator (Inheco, Germany) that is integrated onto the Biomek i7 deck. Tryptic cleavage was stopped by adding 10 µL of 0.22 mg/mL TLCK in 10 mM HCl followed by a 5 min incubation at room temperature. Before the trypsin digested plasma samples were subjected to SISCAPA peptide enrichment, 10 µL of a 10-plex SIL mixture (Supplementary Table 1) was added. The dosing of heavy peptides was optimized during method development to ensure good signal being observed for both disease and healthy controls.

Processing of nasopharyngeal swabs was performed as following, proteins in 180 µL of undiluted Bioer Universal Transport Medium (UTM) were subjected to ice-cold acetone precipitation (7). After centrifugation (16,000g) in a cold environment (0°C) for 10 minutes, the supernatant was discarded and the residual protein pellet was resuspended in 140 µL of a trypsin-Lys C (Promega, Madison, WI, USA) digestion buffer (0.007 µg/µL in 100mM NH_4HCO_3). After a 30-minute incubation at 37°C, trypsin activity was inhibited by adding 20 µL of a 0.22 mg/mL TLCK solution in 10 mM HCl.

Following antibody-bead addition, antibody capture of the target peptides occurs over a period of 1 hour by vigorously shaking the digest plate at 1000 rpm. After incubation, the plates were placed on a magnet array (SISCAPA Assay Technologies) for 90 seconds, to draw the beads to the sides of each well. The supernatant (approximately 185 µL) was removed, and the beads were then washed with 190 µL of wash buffer (10 mM ammonium bicarbonate (ABC), 5% MeOH, 0.00025% CHAPS) followed by resuspending the beads by shaking the plate at 1000 rpm for 1 min. The sample plate was then again placed on the magnet array for 1 minute and 30 seconds before removing the supernatant.

In total, four consecutive washing steps were performed to remove as many unwanted analytes as possible. Subsequently, peptide elution was performed by adding 55 µL of elution buffer (1% formic acid, 0.00025% CHAPS), followed by vigorously shaking for 5 minutes at 1000 rpm.

Solid-Phase Extraction (SPE) clean-up after peptide enrichment *(Optional step for nasopharyngeal swabs)*

Due to the variability and complexity of the nasopharyngeal swab samples, these samples were subjected to an additional solid-phase extraction after peptide elution using the Oasis Hydrophilic-Lipophilic

Balanced (HLB) µElution 96-well plate kit from Waters Corporation (Milford, USA). The HLB plate was conditioned and equilibrated by drawing through 200 µL of respectively, MeOH and water. The peptide enriched samples were loaded onto the sorbent, followed by a wash of 200 µL of 5% MeOH in water. Elution was performed by adding 2 x 10 µL of 40% ACN in 0.1% FA. Finally, the samples were diluted with 35 µL of a 0.5% FA solution in H_20 (0.00025% CHAPS) to increase the total volume and reduce the percentage of ACN. The samples were transferred into an Echo qualified 384-well plate and prior to analysis, the plates were spun at 1000g for 1 minute. Note this step was not required for the enrichment from plasma samples, the samples were desalted well enough after bead washing. The samples were diluted 4x with 0.1% FA to further reduce the organize before LC-MS analysis.

Assessing automation reproducibility

The automation workflow reproducibility was assessed by processing 16 wells of pooled healthy human plasma (two columns on a 96-well plate) and using a pool and split strategy to measure the reproducibility of each portion of the workflow, sample preparation and AEMS. Note that several of the APR proteins are present at low abundance in healthy plasma, therefore an additional amount of synthetic light peptides (CRP (10 pmol), SAA (1 pmol), MBL (1 pmol), MPO (2.5 pmol) and LBP (1 pmol)) were spiked into each of the wells prior to the 30-minute denaturation step.

After sample preparation, the final eluants were transferred to an Echo qualified 384-well plate for MS analysis. To assess reproducibility of the AEMS portion of the workflow, the eluates from one column (eight replicates) were combined and then redistributed into 8 individual samples before analysis. The remaining eight replicates were measured by AEMS individually to determine the overall workflow coefficient of variation (CV). To calculate the imprecision attributable to the automated sample preparation portion of the workflow, the sum of squares model below was employed (17):

$$CV_{i7}^2 = CV_{\text{Total Workflow}}^2 - CV_{MS}^2$$

Supplementary Table 5 shows the breakdown of the %CV for the reproducibility experiment. The total %CV for the light peptides from these 10 proteins with a large dynamic range in plasma ranged from 4.9% to 11.9%. As expected, the variance increases as the area of the ejection peak decreases, so the highest imprecision is observed for the lowest abundant proteins (i.e., CRP and MPO). The imprecision of upstream sample preparation ranged from 2.0% to 7.2%, encompassing both the imprecision arising from digestion variability and the liquid handling imprecision during the SISCAPA procedure. For most of the peptides, the time between ejections was set to 1.5 s, but for SAA a delay time of 3 s was used to ensure good peak separation for all ejections. There was a small amount of tailing in the last ejections, so these data points were removed to allow the computation of the sample preparation reproducibility.

Optimization of AEMS analysis of peptides

Peak shape and intensity of analytes is sensitive to the Acoustic Ejection (AE) conditions, including carrier solvent flow rate and composition and sample ejection volume. These parameters were optimized using a standard peptide mixture (PepCalMix) in a simple matrix (BetaGalactosidase digest) to simulate the

expected complexity of the samples after the SISCAPA enrichment sample preparation. For the carrier solvent composition, various percentage mixtures of water:acetonitrile and water:methanol were tested, along with adding various concentrations of formic acid and medronic acid. The optimal composition was found to be 80% acetonitrile:20% water with 200nM medronic acid.

The flow rate of the carrier solvent was optimized ahead of every project as it is dependent on the aspiration force generated by Gas 1 in the ionization source, the viscosity of the carrier solvent and the cleanliness of the interior of the electrode. Typically for the carrier solvent utilized in these experiments, the flow rate optimized to ~500 µL/min.

To optimize peptide sensitivity, ejection volume optimization focused both on peak area and peak shape for each of the measured PepCalMix peptides. Linearity (r2 = 0.9969 - 0.9994) was observed between the ejection volume (total number of droplets ejected) and MRM peak area from 5 – 400 nL ejections on the three SARS-CoV-2 peptides measured (Supplementary Fig. 2). As the ejection volume increases, the measured peaks get higher in intensity and also increase in peak width at the higher ejection volumes, improving the sensitivity of detection. In the final APR assay, ejection volumes of 100, 200 and 300 nL were used depending on the expected concentration of peptide in the samples. For the SARS-CoV-2 peptide assay, an ejection volume of 300 nL was used for all three peptides.

As AEMS is a flow injection sample introduction strategy, there is no online reverse phase capture and elute as with LC-MS to remove any residual salts from the sample before MS analysis which can, if present result in ion suppression. Additionally, presence of salts can cause tailing of the ejection peaks, necessitating a reduction in the ejection rate, which was deemed undesirable. To characterize the salt tolerance of AEMS for peptide quantification, a variety of salt 1:2 dilution series were created with constant ADETQALPQR peptide concentration (5 fmol/µL) in order to measure the impact of salt on MS signal and ejection peak shape (Supplementary Fig. 3). Additionally, in the immuno-enrichment protocol, CHAPS is often used in the elution buffer to protect the low levels of peptide from binding to plastics, etc (22). Therefore, the concentration of CHAPS that can be present in the sample well without impacting the quality of the ejection peaks or without causing ion suppression was also evaluated. We established a maximum acceptable signal loss of no more than 50% in intensity (Supplementary Fig. 3) to establish elution buffer composition and to inform optimization of washing steps during development of the automation protocol. The starting concentration for each of the tested compounds in the 1:2 dilution series are 0.03% CHAPS, 1x PBS, 100 mM NaCl, 0.2M TRIZMA and 100 mM ABC.

The Echo MS system operates extremely rapidly, ejecting samples into the carrier solvent flow as fast a 1 sample/sec. The observed peak widths are very narrow, on the order of 0.5 seconds wide, depending on the ejection volume. Because both light and heavy transitions for every peptide are monitored and quantitative reproducibility for both areas and L/H area ratios are key, the determination of the optimal acquisition strategy in terms of MRM dwell time and number of MRMs per method is important. Here, a range of dwell times (3 – 100 ms) and number of transitions (2, 4, and 6 MRMs per method) were tested and plotted according to the resulting points across the ejection peak vs. the observed %CV of peak area and L/H peak area ratios (Supplementary Fig. 4). Maintaining 10 or more data points across the ejection peak is critical for maintaining good quantification. We decided to use 4 MRMs per method, 2 per peptide for both light and heavy peptide forms, and 10 ms dwell time for each. An additional MRM for analyzing CHAPS is included in every method (3 ms dwell time) to assist with file splitting, plus the 3 ms pause time between each MRM transition making the total cycle time 58 ms.

Assessing sensitivity of peptide quantification using AEMS

Using the optimized conditions discussed above, concentration curves in simple matrix (0.5% FA, 0.00025% CHAPS) were generated to characterize the typical concentration required in well for peptide detection. Here, PepCalMix (SCIEX) was used because it is a mixture of 20 peptides of various size and hydrophobicity, hence providing a good overall view of peptide sensitivity. Concentration curves from 0.012 – 200 fmol/µL were generated and the lower limit of quantification (LLOQ) for each peptide was determined, using standard bioanalytical criteria for LLOQ of <20% CV and 80-120% accuracy (Supplementary Fig. 5) (23). Good linearity was observed for 19/20 peptides (average R2 of 0.997) and the average LLOQ was 520 amol/µL, with a %CV of 13.4 and an accuracy of 99.7 (Supplementary Table 6). The same protocol was repeated on a second Echo MS system and similar results were observed (LLOQ of 260 amol/µL with %CV of 10.1, accuracy of 104.5%, R2 of 0.988) (Supplementary Table 6). The approximate concentration required in the sample well for good peptide quantification was used to guide the adaptation of the immuno-enrichment protocol for AEMS detection.

Standard addition to estimate the dynamic range of the protein levels

A 14-point standard addition curve was created for the 10-plex to establish the endogenous levels of the ten analytes within pooled human plasma. This curve as shown in Fig. 3A was generated through titration of synthetic light peptides, mirroring the sequences of the endogenous targets, alongside a consistent amount of corresponding heavy isotope-labeled peptides. By assessing the L/H peptide ratio and the known quantity of spiked heavy peptide, the amount of light peptide was computed. The endogenous levels of the analytes, observed by the plateau part in Fig. 3A, encompass a range spanning over six orders of magnitude. As one would expect, even adding 1 nmol of the synthetic light albumin peptide, does not result in a change of signal simply because it is the most abundant circulating protein in plasma, with an approximate concentration of 3.5-5 g/dL (26). Because the antibodies capture the heavy and light peptides with similar affinity, only a small fraction of both forms needs to be captured to calculate an accurate L/H peak area ratio (17). Note that MBL and MPO are the two proteins that exhibit the lowest concentration in pooled healthy plasma, highlighting the challenge in detecting these two proteins using the Echo MS system.
